# The role of wnt signaling in diabetes-induced osteoporosis

**DOI:** 10.1186/s13098-023-01067-0

**Published:** 2023-04-28

**Authors:** Kairan Bao, Yinghua Jiao, Lei Xing, Fang Zhang, Faming Tian

**Affiliations:** 1 Department of Integrated Traditional & Western Medicine, Affiliated hospital of North, China University of Science and Technology, Jianshe South Road 73, Tangshan, 063000 Hebei People’s Republic of China; 2 North China University of Science and Technology, Bohai Road 21, Caofeidian Dis, Tangshan, 063210 Hebei People’s Republic of China

**Keywords:** Diabetes, Osteoporosis, Wnt signaling pathway

## Abstract

Osteoporosis, a chronic complication of diabetes mellitus, is characterized by a reduction in bone mass, destruction of bone microarchitecture, decreased bone strength, and increased bone fragility. Because of its insidious onset, osteoporosis renders patients highly susceptible to pathological fractures, leading to increased disability and mortality rates. However, the specific pathogenesis of osteoporosis induced by chronic hyperglycemia has not yet been fully elucidated. But it is currently known that the disruption of Wnt signaling triggered by chronic hyperglycemia is involved in the pathogenesis of diabetic osteoporosis. There are two main types of Wnt signaling pathways, the canonical Wnt signaling pathway (β-catenin-dependent) and the non-canonical Wnt signaling pathway (non-β-catenin-dependent), both of which play an important role in regulating the balance between bone formation and bone resorption. Therefore, this review systematically describes the effects of abnormal Wnt pathway signaling on bone homeostasis under hyperglycemia, hoping to reveal the relationship between Wnt signaling and diabetic osteoporosis to further improve understanding of this disease.

## Introduction

Diabetes mellitus (DM) is a chronic disease that affects approximately 425 million people worldwide, and research shows that this number will increase by 25% in 2030 and by 51% in 2045 [1]. DM is characterized by long-standing hyperglycemia due to defective insulin secretion, impairment of the biological action of insulin, or both. In addition to chronic damage and dysfunction of the eyes, kidneys, heart, blood vessels, and nerves associated with this disease, recent evidence shows that the risk of osteoporotic fractures in diabetic patients is also significantly increased [2]. Although the prevalence of osteoporosis in diabetic patients is reportedly as high as 60% [3], the insidiousness of this disease means that it is usually detected only after a fracture has occurred. However, the exact etiology of the vulnerability to fragility fractures under hyperglycemia is not fully understood and no effective method to completely prevent or treat this disease has emerged to date. Yet, a growing number of studies have confirmed that disruption of the Wnt signaling pathway is involved in hyperglycemia-induced abnormalities of bone metabolism [4, 5].

The Wnt signaling pathway is critical for regulation of bone metabolic homeostasis and skeletal development [6–8], thus demonstrating its importance in bone remodeling processes. Wnt ligands (Wnt proteins) are a large family of secreted glycoprotein ligands (encoded by *Wnt* genes) that activate canonical or non-canonical Wnt signaling pathways by binding related receptors, such as frizzled (FZD), low-density lipoprotein receptor-related protein 5/6 (LRP5/6), receptor-tyrosine-kinase-like orphan receptor 1/2 (ROR1/2), and receptor-like tyrosine kinase (RYK) [9–11]. At present, 19 distinct Wnt ligands have been identified in vertebrates, each with a varying degree of influence on different stages of skeletal development (i.e., chondrogenesis, osteoblastogenesis, and osteoclastogenesis) [12]. Among these Wnt ligands, Wnt2b, Wnt3, Wnt8a, Wnt8b, Wnt9a, and Wnt10b mainly activate the canonical Wnt signaling pathway; Wnt11 activates the non-canonical Wnt signaling pathway; and Wnt1, Wnt2, Wnt3a, Wnt4, Wnt5a, Wnt5b, Wnt6, Wnt7a, Wnt7b, Wnt9b, Wnt10a, and Wnt16 act as promoters of both signaling pathways [13–15]. Wnt signaling pathways are mainly divided into two categories: canonical (β-catenin-dependent) and non-canonical (non-β-catenin-dependent). In addition to the Wnt ligands that activate each pathway, the canonical pathway includes antagonist factors, such as Dickkopf (DKK) and sclerostin (SOST) proteins, that can bind LRP5/6 to inhibit the function of LRP5/6 and FZD [16]. In contrast, as decoy receptors of Wnt ligands, secreted frizzled-related proteins (SFRPs) and Wnt inhibitory factor 1 (WIF1) can directly bind to Wnt [17] to inhibit both canonical and non-canonical Wnt signaling pathways.

The Wnt signaling pathway plays a key role in regulating the balance between osteoblasts and osteoclasts. Disruption of either the canonical or non-canonical Wnt signaling cascade can severely interrupt the normal bone remodeling process [18]. In general, the degree of bone formation and resorption is equal, and any pathological factor that disrupts this coupling will lead to bone remodeling disorders, such as osteoporosis. However, high glucose can inhibit the activity of osteoblasts, increase the activity and number of osteoclasts, weaken the differentiation of bone marrow-derived mesenchymal stem cells (BMSCs) into osteoblasts, and enhance the generation of adipocytes, resulting in a reduction of bone mass [19–21]. It is thus concluded that long-term hyperglycemia inevitably disrupts the Wnt signaling pathway to a certain extent, which eventually causes bone resorption to be greater than bone formation, leading to osteoporosis. Accordingly, investigating roles of Wnt signaling in diabetic osteoporosis will help us further understand its pathogenesis and provide new strategies for the treatment of this disease. The canonical Wnt signaling pathway is widely studied and has great potential therapeutic value for the treatment of osteoporosis by promoting bone formation. However, constitutive activation of β-catenin has been shown to increase the risk of cancer [22]. Therefore, this review also describes roles of the non-canonical Wnt signaling pathway with the goal of providing a more comprehensive understanding of the effects of high glucose on bone homeostasis through Wnt signaling.

## Canonical wnt signaling pathway

**Fig. 1:**
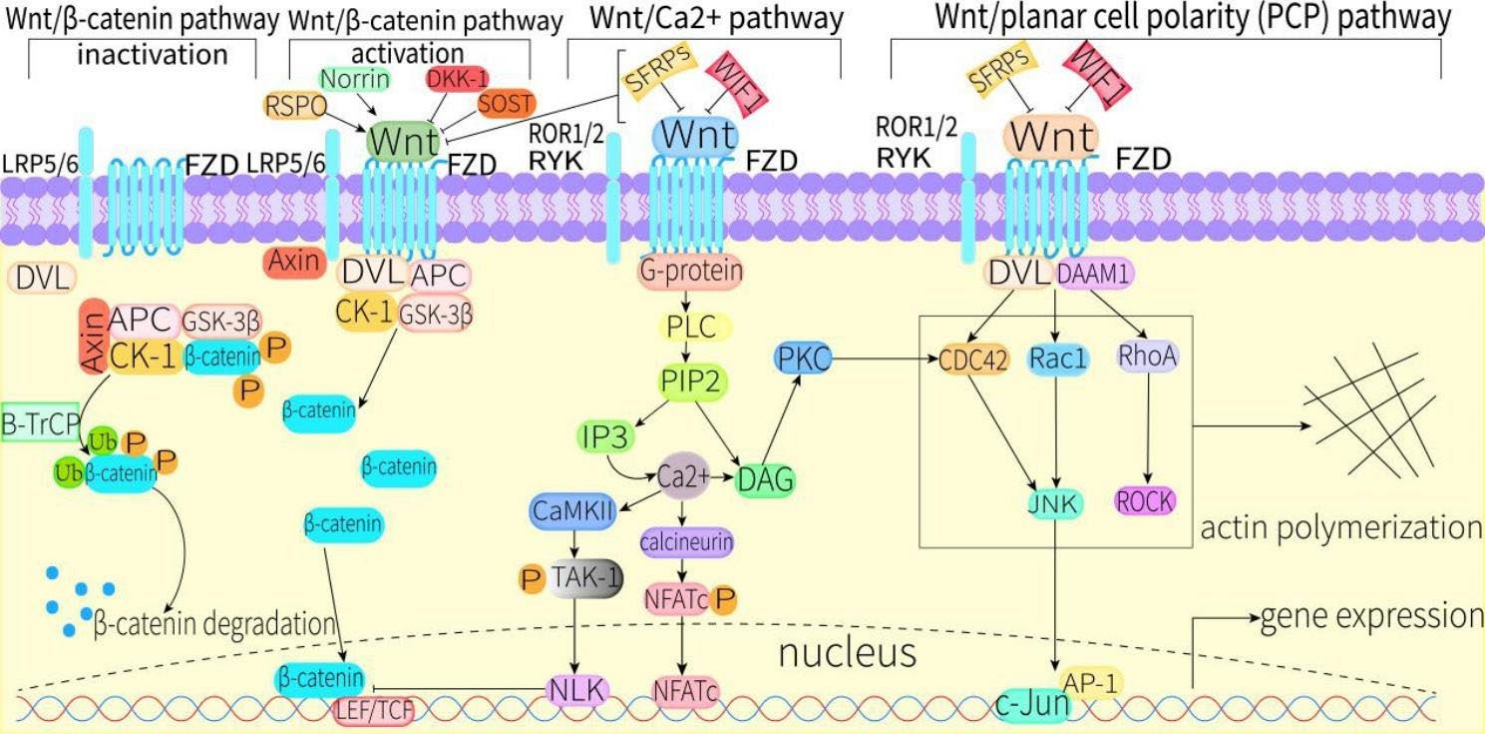
Canonical and non-canonical Wnt signaling pathways under normal conditions. Abbreviations: APC, adenomatous polyposis; AP-1, activator protein 1; B-TrCP, β-transducin repeat-containing protein; CK-1, casein kinase 1; CaMKII, calmodulin-dependent protein kinase II; CDC42, cell division cycle 42; c-Jun, Jun proto-oncogene; DAAM1, dishevelled associated activator of morphogenesis 1; DVL, dishevelled; DKK-1, Dickkopf-1; DAG, 1,2 diacylglycerol; FZD, frizzled; GSK-3β, glycogen synthase kinase-3β; IP3, inositol-1,4,5-trisphosphate; JNK, c-Jun N-terminal kinase; LRP5/6, low-density lipoprotein receptor-related protein5/6; LEF/TCF, lymphoid enhancer factor/T cell factor; NLK, Nemo-like kinase; NFATc, nuclear factor-activated T cell; Norrin, Norrie Disease gene product; P, phosphorylation; PIP2, phosphatidylinositol-4,5-bisphosphate; PKC, protein kinase C; PLC, phospholipase C; RSPO, R-spondin proteins; RYK, receptor like tyrosine kinase; ROR1/2, receptor-tyrosine-kinase-like orphan receptor 1/2; Rac1, Rac family small GTPase 1; RhoA, Ras homolog family member A; ROCK, RHO-associated kinase; SOST, sclerostin; SFRPs, secreted frizzled-related proteins; TAK-1, transforming growth factor (TGF)-β -activated kinase 1; Ub, polyubiquitination; WIF1, Wnt inhibitory factor 1;

In the absence of Wnt ligands, β-catenin is phosphorylated by a degradation complex composed of Axin, adenomatous polyposis (APC), casein kinase 1 (CK-1), and glycogen synthase kinase 3β (GSK-3β). Subsequently, phosphorylated β-catenin is degraded via the ubiquitin-proteasome pathway. Once a canonical Wnt ligand binds to the dual receptor complex composed of FZD and LRP5/6, the Wnt/β-catenin signaling pathway is activated. Axin subsequently moves to the tail of LRP5/6, while dishevelled (DVL) is recruited by FZD to form a complex with APC, CK-1, and GSK-3β, leading to the accumulation of β-catenin in the cytoplasm [23, 24]. The accumulated β-catenin translocates to the nucleus, whereby it interacts with lymphoid enhancer factor/T cell factor (LEF/TCF) to stimulate the expression of target genes [25, 26]. As agonists of this pathway, Norrie Disease gene product (Norrin) and R-spondin proteins can enhance the canonical Wnt signaling pathway [27]. Studies have confirmed that Wnt/β-catenin signaling is active in various osteoblast and osteoblast precursor cell lines, and can promote bone formation when activated in these cells [28, 29] (Fig. 1).

## Effect of high glucose on the canonical wnt signaling pathway in bone homeostasis

### Ligands

Wnt1/β-catenin signaling, Wnt3a/β-catenin signaling and Wnt10b/β-catenin signaling axes are critical for the transition of BMSCs [or mouse embryo osteoblast precursor cells (MC3T3-E1 cells)] to the osteoblasts [30–35]. Conditional expression of Wnt1 under physiological conditions rapidly increases the number and function of osteoblasts, thus causing a rapid increase in bone mass [36, 37]. Wnt1 inhibits osteoclastogenesis via canonical Wnt signalling in a mouse monocyte-macrophage leukemia cell line (RAW264.7 cells) [38]. Wnt3a, an important target of bone remodeling, increased the secretion of osteoprotegerin [OPG, a decoy receptor of receptor activator of nuclear factor κB ligand (RANKL)] by activating the Wnt/β-catenin signaling pathway to accelerate bone repair processes in osteoporosis models; whereas, silencing of Wnt3a reduced osteoblast differentiation and mineralization [39, 40]. Wnt3a can directly suppresses osteoclast differentiation through canonical Wnt signalling in bone marrow monocyte-macrophage lineage cells (BMMs) [41]. Stevens et al. found that deletion of Wnt10b in mice caused a decrease in the number of mesenchymal progenitor cells rather than altered osteoclast activity or number [42], indicating that Wnt10b functions predominantly on mesenchymal or osteoblast progenitor cells. Increasing expression of Wnt10b was shown to improve glucose homeostasis and reduce plasma triglyceride levels in obese rats [43]. Peroxisome proliferator-activated receptorγ (PPARγ) and CCAAT/enhancer binding proteinα(C/EBPα) are the ultimate regulators in the differentiation of BMSCs to adipocytes, whereas PPARγ and C/EBPα are in a suppressed state when the canonical Wnt signaling pathway is activated [44]. Notably, Wnt1, Wnt3a and Wnt10b are endogenous inhibitors of adipocytogenesis [45–47].

**Fig. 2:**
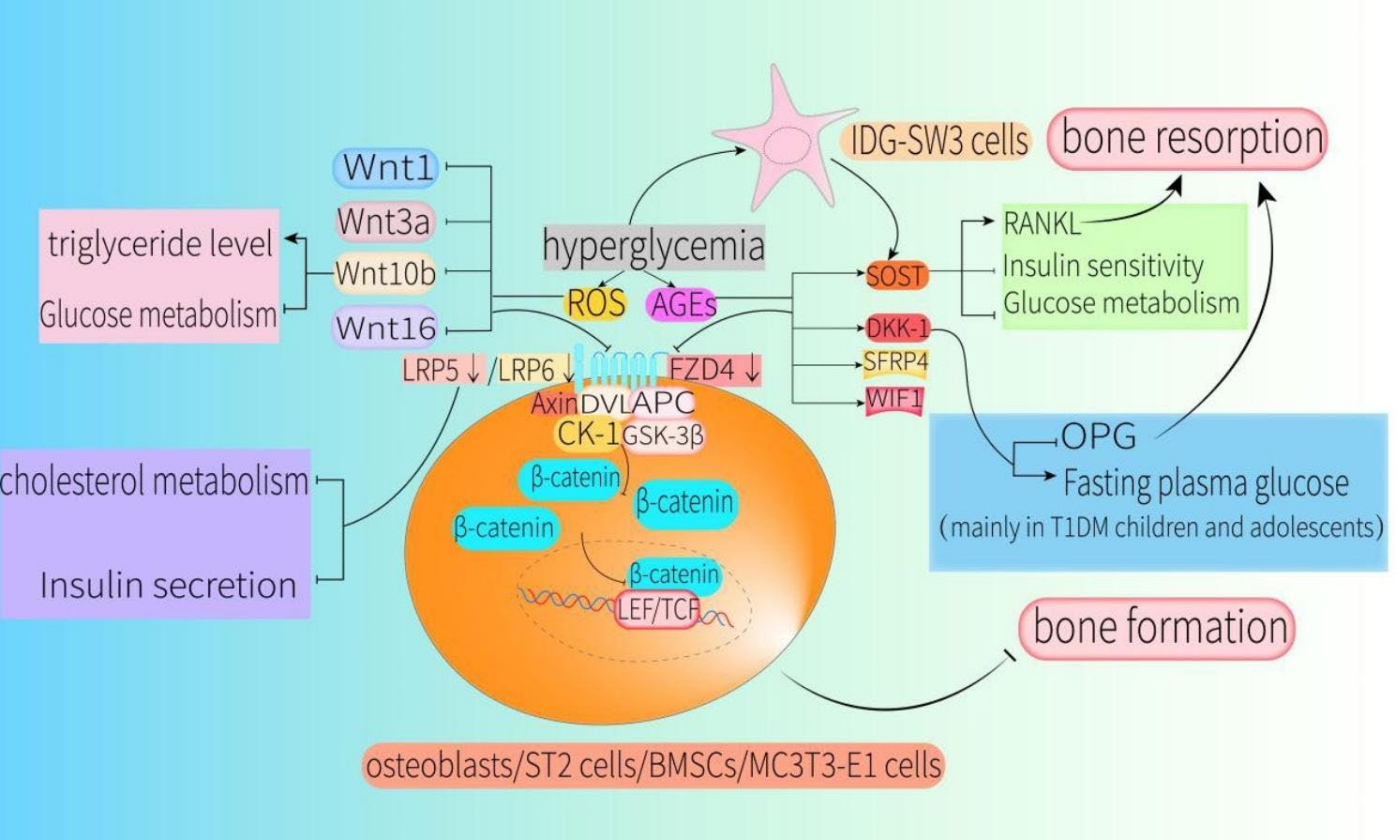
Hyperglycemia affects bone formation, bone resorption, glucose metabolism, and lipid metabolism by disrupting the canonical Wnt pathway in osteoblasts, ST2 cells, BMSCs, MC3T3-E1 cells, and IDG-SW3 cells Hyperglycemia-generated ROS and AGEs can inhibit the Wnt/β-catenin signaling pathway in a manner that inhibits expression of Wnt ligands and receptors, and increases expression of antagonists, thereby inhibiting bone formation and increasing bone resorption. In particular, decreases in Wnt10b and LRP5 affect glucose and lipid metabolism, as well as insulin secretion. Elevated glucose can directly act on IDG-SW3 cells to increase SOST expression, leading to increased RANKL expression, decreased glucose metabolism, and reduced insulin sensitivity. Simultaneously, increased levels of DKK-1 caused by high glucose will inhibit OPG secretion by osteoblasts and increase fasting plasma glucose levels (mainly in children and adolescents with T1DM) Abbreviations: ST2cells, bone marrow stromal cells of ST2 mice; BMSCs, bone mesenchymal stem cells; MC3T3-E1 cells, mouse embryo osteoblast precursor cells; IDG-SW3 cells, osteocyte-like cell line; ROS, reactive oxygen species; AGEs, advanced glycation end products; RANKL, receptor activator of nuclear factor κB ligand; OPG, osteoprotegerin

**Fig. 3:**
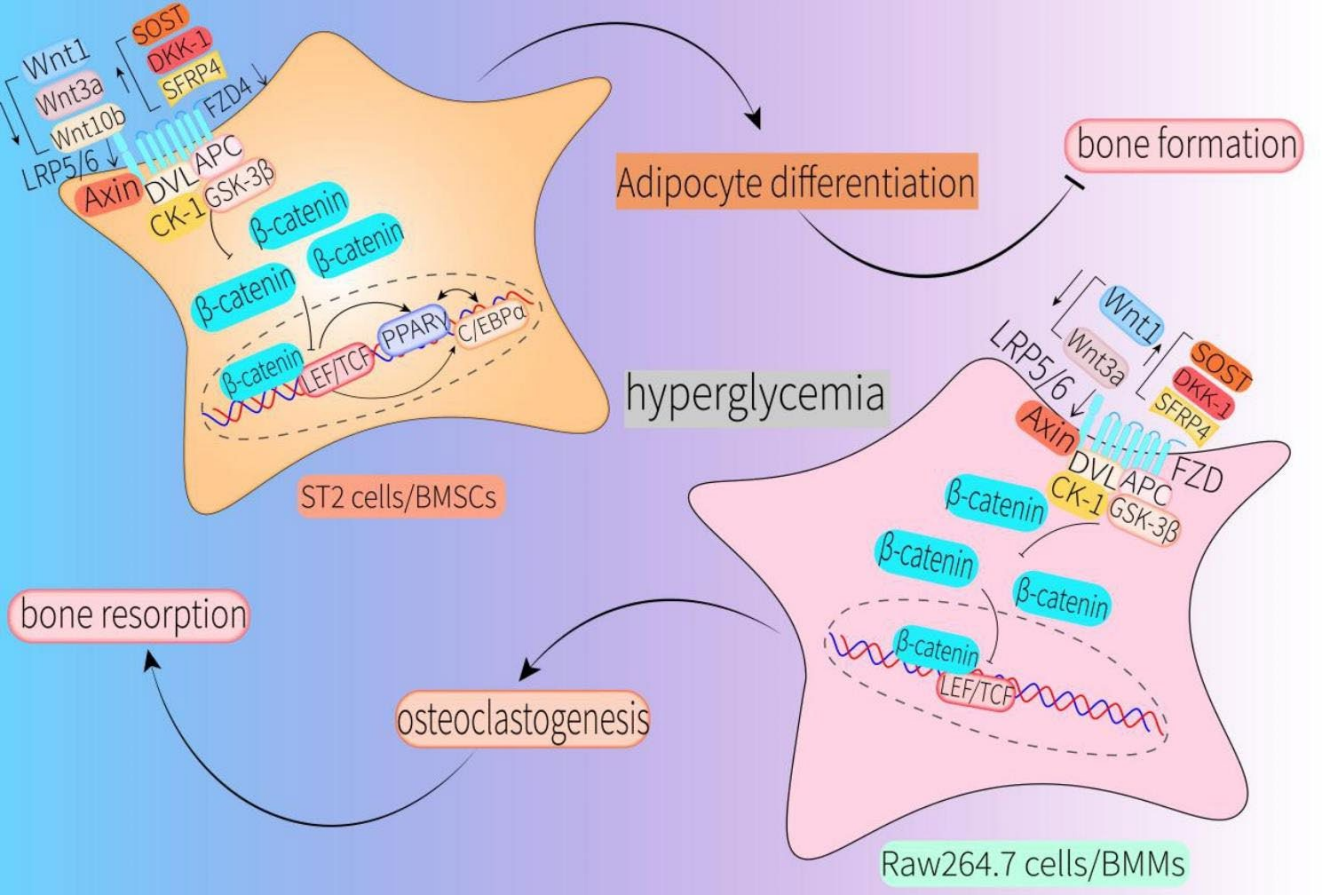
Hyperglycemia promotes adipocytogenesis in ST2 cells and BMSCs, as well as stimulates osteoclastogenesis in RAW264.7 cells and BMMs, by inhibiting canonical Wnt signaling Hyperglycemia-induced decreased expression of Wnt1, Wnt3a, Wnt10b, LRP5/6 and FZD4, and increased expression of SOST, DKK-1 and SFRP4, jointly inhibited canonical Wnt signaling in ST2 cells and BMSCs, resulting in the activation of PPARγ and C/EBPα, which triggered increased adipocytogenesis and inhibited bone formation. Hyperglycemia-induced decreased expression of Wnt1, Wnt3a and LRP5/6, and increased expression of SOST, DKK-1 and SFRP4, together inhibited canonical Wnt signaling in RAW264.7 cells and BMMs, which provoked increased osteoclastogenesis and promoted bone resorption Abbreviations: PPARγ, Peroxisome proliferator-activated receptorγ; C/EBPα, CCAAT/enhancer binding proteinα; RAW264.7 cells, mouse monocyte-macrophage leukemia cell line; BMMs, bone marrow monocyte-macrophage lineage cells

However, Shao et al. found that expression of Wnt1, Wnt3a, and Wnt10b in a mouse model of type 2 diabetic osteoporosis established with streptozotocin was significantly lower compared with that in control groups. Moreover, this study found that chronic hyperglycemia and production of advanced glycosylation end products (AGEs) were the main causes of the observed adverse effects [48]. This implies that a decrease in Wnt1, Wnt3a and Wnt10b caused by high glucose can lead to a decrease in osteoblastogenesis, an increase in osteoclastogenesis and adipocytogenesis through canonical Wnt signaling (Figs. 2 and 3).

High dose of Wnt16 can not only promote the expression of OPG by activating the canonical Wnt signaling pathway in osteoblasts, but also accelerate the differentiation process toward osteoblasts by activating the WNT16/β-catenin axis in BMSCs or MC3T3-E1 cells [49–51]. In contrast to the findings in osteoblasts, Movérare-Skrtic et al. found that Wnt16 did not significantly regulate β-catenin protein levels or Axin2 expression in mouse osteoclast precursors [52]. Nevertheless, we have not found researches on how Wnt16 affects the differentiation of BMSCs to adipocytes. However, Chen et al. found that Wnt16 expression was significantly reduced in cortical bone of type 1 diabetes mellitus (T1DM) mice. Indeed, this was the main reason for the unexpected increase in cortical porosity and bone resorption in the context of T1DM, and AGEs and oxidative stress were involved in these abnormal changes [53]. This implicates that a decrease in Wnt16 expression can lead to a decrease in osteoblastogenesis through canonical Wnt signaling (Fig. 2).

### Receptors

In the physiological state, the two receptors LRP5 and LRP6 are not completely redundant, and both must be expressed in osteoblast lineage for normal bone development. Reducing LRP5/6 protein expression prevents Wnt3a-suppressed differentiation of BMMs to osteoclasts [41]. Augmentation of LRP5/6 expression prevents the differentiation of bone marrow stromal cells of ST2 mice (ST2 cells) into adipocytes [54, 55]. However, Wang et al. found that expression of LRP5 and OPG was significantly reduced in fractures of T1DM rats compared with that in fractures of rats without concomitant diabetes, and bone remodeling could be significantly promoted by inhibiting DKK-1 and improving islet function in this model [56]. Cai et al. found that LRP6 expression was significantly reduced in bone defects of a rabbit model of T1DM compared with that in controls, and confirmed involvement of hyperglycemia-induced inhibition of the canonical Wnt signaling pathway in abnormal bone turnover in T1DM [57].

In addition, promotion of LRP5 expression increased bone mass and bone strength in insulin-deficient diabetic mice, delaying the onset of hyperglycemia in these mice [58]. LRP5 is also essential for normal cholesterol metabolism and glucose-induced insulin secretion. For example, mice specifically lacking LRP5 in osteoblasts and osteocytes exhibited reduced energy expenditure and increased body fat [59]. Male mice with *Lrp5* deletion in osteoblasts fed a high-fat diet also showed strong tendencies for hyperglycemia and insulin resistance [60].

FZD is another key receptor involved in canonical Wnt signaling. Kushwaha et al. found that FZD4-deficient mice exhibited a significant decrease in cortical bone mineral density (BMD) and impaired femoral trabecular formation despite a simultaneous increase in FZD8 expression, suggesting that FZD4 remains necessary for normal bone remodeling even in the presence of FZD8 compensation [61]. However, Wang et al. determined that FZD4 expression in BMSCs of diabetic rats was significantly reduced compared with that in controls and triggered the impairment of bone formation. Moreover, the inhibitory effect of hyperglycemia on bone formation was significantly alleviated by targeting expression of FZD4 and β-catenin [62] .

**Fig. 4:**
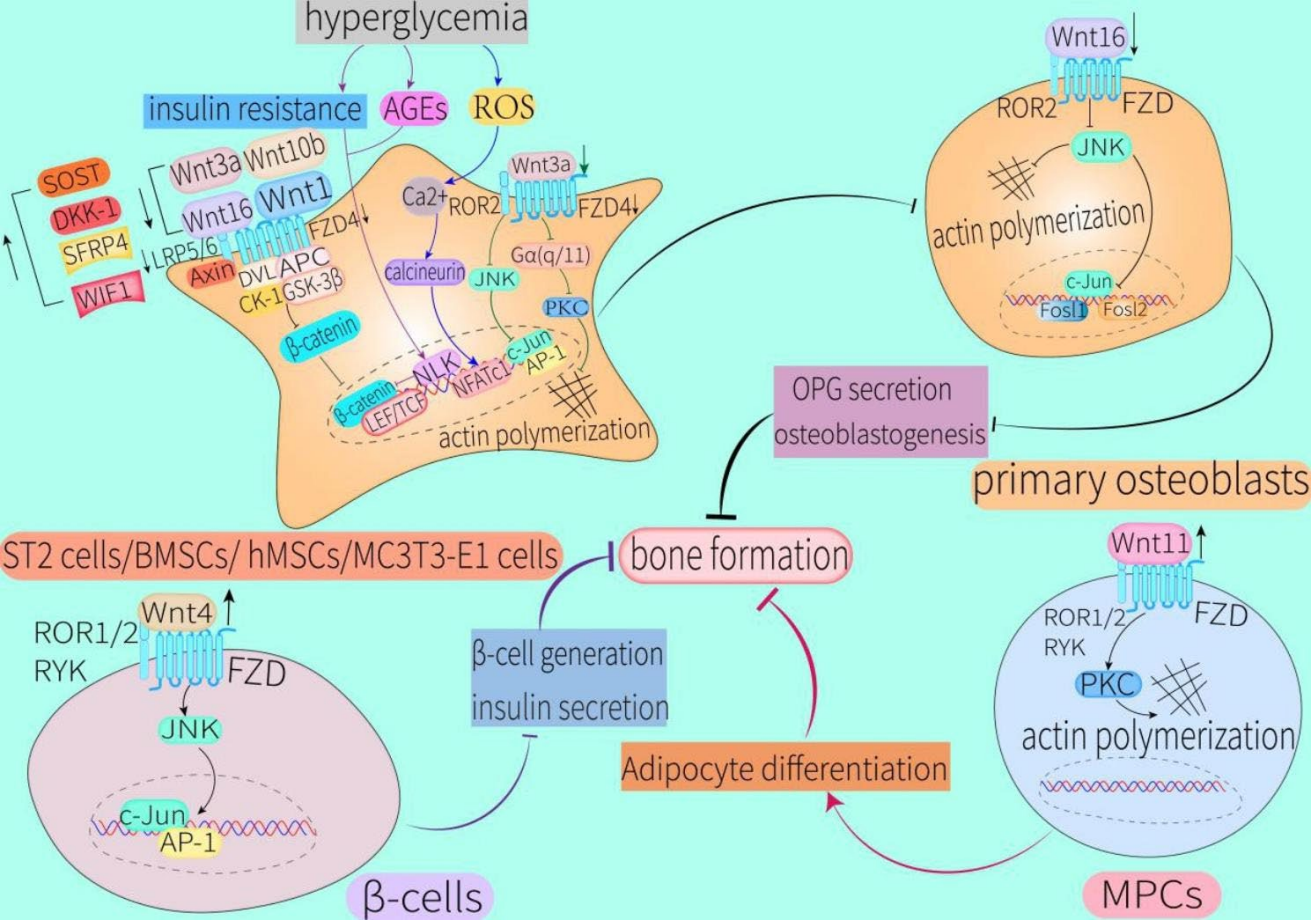
Effects of hyperglycemia on osteoblast precursors, primary osteoblasts, and β cells via the non-canonical Wnt pathway In ST2 cells, BMSCs, hMSCs or MC3T3-E1 cells: hyperglycemia-induced insulin resistance and production of AGEs can directly lead to the activation of NLK, which works in conjunction with the reduction of Wnt ligands, LRP5/6, and FZD4, and increase of Wnt antagonists to inhibit the Wnt/β-catenin pathway. Hyperglycemia-generated ROS can negatively regulate the differentiation process of these cells towards osteoblasts by activating the Ca^2+^/calcineurin/NFATc1 pathway. Hyperglycemia-induced reductions in Wnt3a expression inhibit actin polymerization via the Gα(q/11) (heterotrimeric G protein)/PLC/PKC pathway or JNK pathway, thereby affecting morphological maturation of these cells. In primary osteoblasts: hyperglycemia inhibits the JNK pathway by reducing Wnt16 expression, thereby inhibiting actin polymerization and preventing the binding of c-Jun to transcription factors (e.g. Fosl1 and Fosl2) to inhibit the maturation of primary osteoblasts into mature osteoblasts. In β cells: hyperglycemia-induced increases in Wnt4 levels decrease β cell formation and insulin secretion by activating the JNK pathway in these cells. In MPCs: hyperglycemia-induced increases in Wnt11 promote differentiation of these cells toward adipocytes rather than osteoblasts by activating PKC. Taken together, hyperglycemia can ultimately inhibit bone formation through the above pathways Abbreviations: hMSCs, human mesenchymal stem cells; MPCs, human bone marrow mesenchymal progenitor cells; Fosl1, recombinant FOS like-antigen 1; Fosl2, recombinant FOS-like antigen 2

**Fig. 5:**
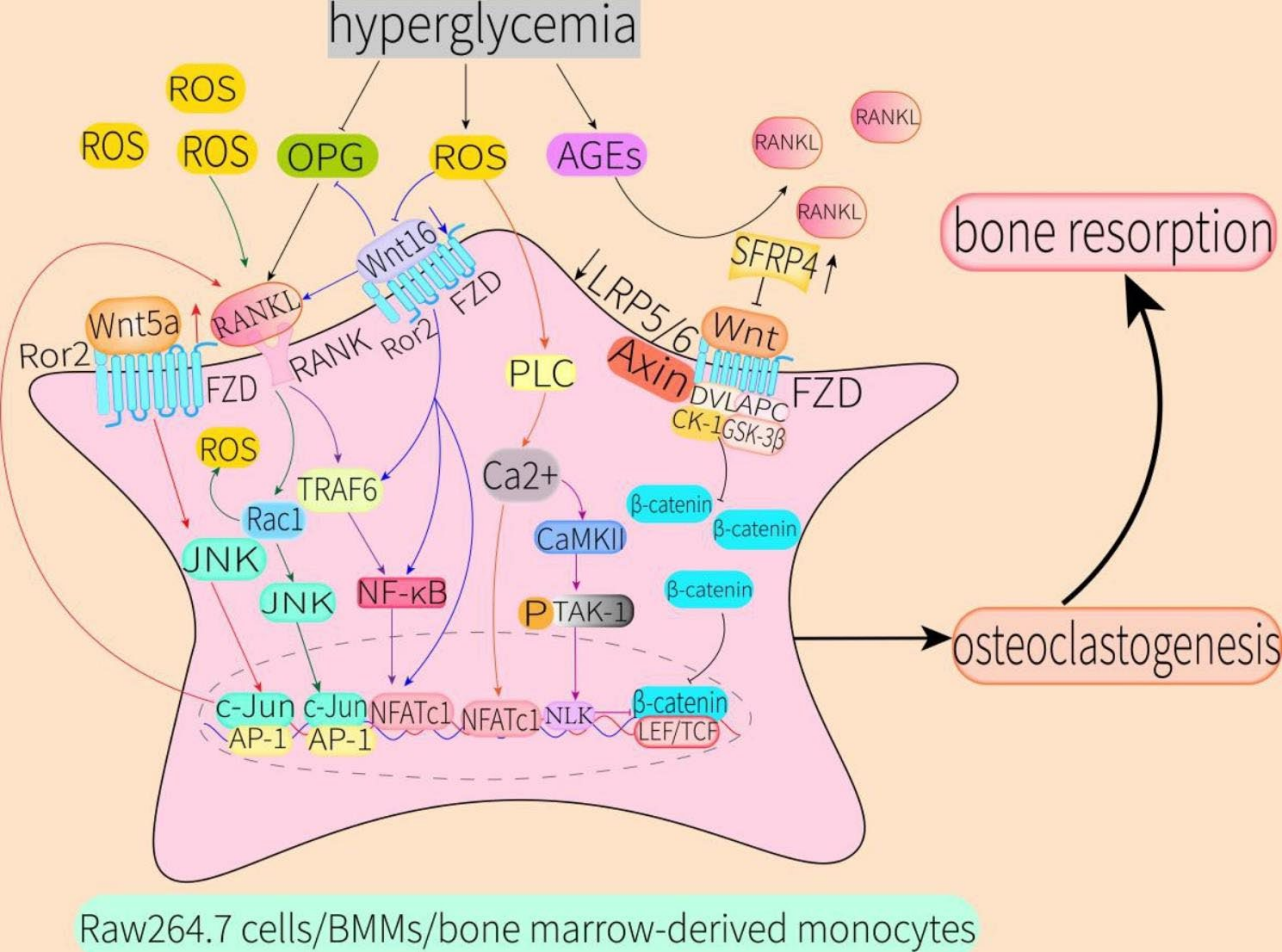
Effect of hyperglycemia on differentiation of RAW264.7 cells, BMMs, and bone marrow-derived monocytes into osteoclasts through the non-canonical Wnt signaling pathway Hyperglycemia can increase Wnt5a expression, thereby causing JNK pathway activation to promote binding of RANKL and RANK. The combination of RANKL and RANK can activate the Rac1/JNK pathway and TRAF6/NF-κB/NFATC1 pathway to promote osteoclast differentiation, while activation of Rac1 can lead to ROS production. ROS produced by hyperglycemia can also inhibit expression of Wnt16, which can lead to increased activity of TRAF6, NF-κB, and NFATc1 to further activate the TRAF6/NF-κB/NFATC1 pathway. The decrease of Wnt16 level also increases binding of RANKL and RANK to inhibit expression of OPG. In addition, hyperglycemia can inhibit OPG secretion. As the main decoy receptor of RANKL, decreased OPG levels can further enhance binding of RANKL and RANK to promote osteoclastogenesis. ROS produced by hyperglycemia can directly activate the PLC/Ca2+/NFATc1 pathway in the resulting osteoclast precursors, which has a positive feedback effect on osteoclastogenesis. Activation of the Wnt/β-catenin signaling pathway inhibits differentiation of osteoclast precursors into osteoclasts. However, hyperglycemia-induced increased expression of SFRP4 and decreased expression of LRR5/6 collectively lead to inhibition of the Wnt/β-catenin signaling pathway in these monocytes. In addition, ROS produced by hyperglycemia can induce activation of the Ca^2+^/CaMKII/TAK-1/NLK pathway in osteoclast precursors. Therefore, increased SFRP4 expression, decreased LRR5/6 expression, and activation of the Ca^2+^/CaMKII/TAK-1/NLK pathway could further inhibit the Wnt/β-catenin signaling pathway in osteoclast precursors, resulting in increased osteoclastogenesis. In summary, hyperglycemia can lead to enhanced bone resorption through the above pathways Abbreviations: RANK, receptor activator of nuclear factor-κB; TRAF6, tumor necrosis factor-associated factor 6; NF-κB, nuclear factor-κB

From the above studies, we can confirm that high glucose reduces the expression of LRP5/6 in diabetic individuals to inactivate canonical Wnt signaling, which subsequently inhibits osteoblastogenesis, increases osteoclastogenesis and adipocytogenesis. However, inhibition of osteoblastogenesis due to reduced FZD4 expression caused by high glucose is currently only seen in mesenchymal stem cells (Figs. 2, 3, 4 and 5).

### Β-catenin

As a key component of the canonical Wnt signaling pathway, β-catenin is required for postnatal osteoblast differentiation and proliferation, and essential for the anabolic effect of parathyroid hormone in bone [63]. β-catenin deletion at committed stages of osteoclast differentiation will result in osteoporosis because of enhanced osteoclast differentiation [41]. Pharmacological treatments that activate β-catenin in BMSCs can trigger osteoblastogenesis and prevent adipogenesis [64]. However, in an analysis of 40 postmenopausal women with type 2 diabetes mellitus (T2DM) and 40 healthy controls, Gaudio et al. found that β-catenin levels were significantly lower in women with T2DM compared with those in the control group, which may be related to the production of AGEs and increase of SOST [65]. Razny et al. demonstrated that the expression of Wnt pathway antagonists DKK, SOST, SFRPs, and WIF1 was significantly upregulated in obese individuals with T2DM and insulin resistance, while expression of β-catenin (a key factor of osteoblast formation) was significantly decreased [66].

Inhibition of the canonical Wnt signaling pathway mainly targets the degradation of β-catenin. High glucose can also significantly decrease levels of β-catenin in MC3T3-E1 cells by inducing the production of reactive oxygen species (ROS) and AGEs [67]. However, Li et al. found that inhibition of GSK-3β activity in BMSCs of diabetic osteoporotic rats could activate β-catenin and relieve the inhibition of osteoblast differentiation under hyperglycemia [68]. In addition, Xiong et al. demonstrated that osteoblasts treated with Wnt3a could alleviate the inhibition of β-catenin caused by high glucose, and 1,25(OH)_2_D_3_ could promote β-catenin transfer from forkhead transcription factor O1 to Wnt/TCF-mediated transcription under high glucose [69].

Collectively, these researches indicate that inhibition of β-catenin activity in diabetic individuals will contribute to reduced bone formation, increased bone resorption, and enhanced adipogenesis. Thus β-catenin is a key factor in reversing the inhibitory state of canonical Wnt signaling (Figs. 2 and 3).

### SOST

Specifically secreted by osteocytes, SOST has an antianabolic effect on bone formation and inhibits osteoblast activity to downregulate bone turnover rates [70, 71]. Reduction of the SOST gene can promote bone formation through Wnt/β-catenin signaling and compensate for particle-induced osteolysis [72]. Inhibition of SOST expression resulted in a dramatic reduction in the number of osteoclasts in cancellous bone, thus causing an increase in cancellous bone mass [73]. Sclerostin also attenuated canonical Wnt3a-inhibited adipocyte differentiation [74], showing that SOST is a promotive factor for adipocytogenesis. However, serum levels of SOST are significantly increased in patients with T2DM and femur fractures [75], suggesting that hyperglycemia may increase the expression of SOST by antagonizing canonical Wnt signaling to cause bone resorption beyond bone formation and increase adipocytogenesis. In addition, the osteoclastogenic factor RANKL was positively correlated with SOST expression [76]. Neumann et al. found that serum SOST levels were significantly increased in adults with T1DM compared with those in controls, and a positive correlation between age and serum SOST level was more obvious in these patients [77]. Tsentidis et al. confirmed that SOST was positively correlated with bone formation and resorption markers in children and adolescents with T1DM [78]. Singh et al. found that serum SOST levels were higher in individuals with pre-diabetes and strongly associated with systemic insulin sensitivity, fasting endogenous glucose production, and insulin clearance rate [79]. IDG-SW3 cells (an osteocyte-like cell line) cultured under high glucose conditions displayed significantly increased SOST expression compared with the control group [80].

In conclusion, these results suggest that SOST not only modulates the homeostasis of bone metabolism, but it is crucial for the regulation of human glucose metabolism. Thus, SOST is clearly important for the processes underlying diabetic osteoporosis and deserves greater investigation in DM-induced bone metabolic dysfunction to further elucidate its pathogenic roles (Figs. 2 and 3).

### Dkk-1

DKK-1, another antagonist of the Wnt/β-catenin signaling pathway, is predominantly expressed by osteocytes, although not as highly or selectively as SOST. DKK-1 is an important regulator of bone mass, but its overexpression can impair bone regeneration [81]. Heiland et al. found that the number of osteoclasts was significantly reduced in mice treated with anti-DKK-1 antibody [82], suggesting that DKK-1 is a positive regulator of osteoclastogenesis. Knockdown of DKK-1 abrogated dexamethasone-induced accumulation of cytoplasmic oil droplets and the number of adipocyte-like cells in cultured mouse mesenchymal progenitors [83], suggesting that DKK-1 is also a positive regulator of adipocytogenesis. In addition, when DKK1 and RANKL levels were significantly and simultaneously increased, osteoblast differentiation and OPG expression were more inhibited in patients with osteogenesis imperfecta with higher DKK-1 levels [84], demonstrating that DKK-1 has a more detrimental effect on bone formation.

However, in children and adolescents with T1DM, DKK-1 levels were more highly expressed compared with those in controls, thus causing a significant inhibition of the Wnt/β-catenin pathway. The result is reduced osteoblast activation and increased osteoclastogenic factors, which are responsible for reduced bone turnover in individuals with diabetes [85], implicating that hyperglycemia can have a direct negative effect on bone metabolism in young patients by increasing DKK-1 levels. In addition, DKK-1 is positively correlated with fasting plasma glucose, but is mainly seen in children and adolescents with T1DM [85, 86]. In a comparison of 21 women with T2DM and 21 women without diabetes as controls, Sassi et al. found a lower bone turnover rate and significantly higher DKK-1 in women with T2DM [87], indicating that poor glycemic control may interfere with the balance of cytokines associated with normal bone turnover. A significant increase in DKK-1 has also been found in patients with T2DM combined with cardiovascular disease, while a correlation between DKK-1 and vertebral fractures has not been demonstrated in these individuals. In addition, Wnt/β-catenin signaling in a T1DM mouse model induced by streptozotocin was significantly downregulated compared with that in the control group, which was confirmed by increased DKK-1 levels and decreased β-catenin levels in the proximal tibia [88]. Although knockdown of the Dkk-1 gene in osteoblasts did not alter the metabolic parameters of T1DM, it did partially prevent trabecular bone defects and completely prevent cortical bone damage caused by T1DM [89]. Previous studies have also found positive correlations between DKK-1 and SOST in individuals with T2DM [90].

From the findings above, it can be confirmed that DKK-1 is an important negative regulator of bone formation. Increased DKK-1 can trigger increased bone loss and bone marrow adipogenesis in DM and is closely associated with reduced glucose metabolism in children and adolescents with T1DM (Figs. 2 and 3).

### SFRPs and WIF1

SFRPs can directly bind to Wnts to block the transmission of Wnt signaling. Notably, the role of SFRP4 in diabetic osteoporosis is perhaps the most unique. Impaired osteoblast differentiation was accompanied by upregulation of the Wnt signaling antagonist SFRP4 and impairment of Wnt signaling [91]. The loss of canonical Wnt signaling pathway inhibition in SFRP4-KO mice caused increases in bone trabecular bone mass [92]. Enhanced adipogenic differentiation can be inhibited in ST2 cells by silencing SFRP4 to activate canonical Wnt signaling [93], indicating that SFRP4 is a promoter of adipocytogenesis. The increase of bone fragility induced by oxidative stress caused by diabetes and aging is also partly related to the inhibition of Wnt signaling through upregulation of SFRP4 [94]. In addition, Diabetic osteoporosis caused by decreased Wnt/β-catenin signaling is partly induced by epigenetic derepression of the SFRP-4 gene [95]. Mastaitis et al. found that diet-induced obese SFRP4-KO mice exhibited reduced energy expenditure, food intake, and BMD, but had similar glucose tolerances to control mice; moreover, SFRP4 overexpression did not affect energy metabolism or glucose homeostasis [96]. Because SFRP-4 is a soluble factor, the use of neutralizing antibodies or inhibitors could be a good therapeutic strategy for diabetic osteoporosis [95]. Altogether, these results indicate that hyperglycemia-induced elevation of SFRP4 leads to an imbalance in bone remodeling and increased adipocytogenesis, rather than glucose metabolism. Therefore, SFRP4 may be a good target for the treatment of diabetic osteoporosis (Figs. 2 and 3).

Like SFRPs, WIF1 also acts as a decoy receptor and inhibits canonical Wnt signaling. The WIF1/Wnt/β-catenin pathway plays an essential role in osteogenic differentiation [97]. Morimoto et al. found that glucocorticoid-mediated inhibition of osteoblast growth and reduction of differentiation of ST2 cells to osteoblasts could be alleviated by silencing WIF1 [98], demonstrating that WIF1 is an inhibitory factor of osteoblastogenesis. WIF1 is elevated in individuals with T2DM [66]. Nevertheless, currently there is a lack of WIF1 effects on osteoclastogenesis and adipocytogenesis through canonical Wnt signaling. However, it is at least certain that elevation of WIF1 in T2DM individuals inhibits osteoblast differentiation and growth via canonical Wnt signaling (Fig. 2).

Altogether, the results described above demonstrate that in addition to affecting bone homeostasis, the Wnt/β-catenin signaling pathway is closely related to endocrine metabolism. Moreover, the Wnt/β-catenin signaling pathway is significantly inhibited in the high glucose state, which leads to reduced bone formation, enhanced bone resorption, elevated bone marrow adipogenesis, inhibited insulin secretion, and reduced glucose and lipid metabolism.

## Non-canonical wnt signaling pathway

The non-canonical Wnt signaling pathway is involved in morphological maturation caused by osteoblast and osteoclast differentiation because it plays important roles in the regulation of cell motility, cell polarity, and cytoskeleton remodeling [99]. The non-canonical Wnt signaling pathway can be divided into the Wnt/planar cell polarity (PCP) signaling pathway and Wnt/Ca^2+^ signaling pathway. In the Wnt/PCP signaling pathway, a complex formed by FZD and RYK or ROR1/2 binds to Wnt proteins and co-recruits DVL, which then binds to dishevelled associated activator of morphogenesis 1 (DAAM1) to co-activate Rac family small GTPase1 (RAC1), RAS homolog family member A (RhoA), and cell division cyclin 42 (CDC42). Activation of these Rho family small GTPases is involved in the regulation of cytoskeleton remodeling, actin polymerization, cell polarization, and cell motility. Among them, Rac1 and CDC42 can co-activate c-Jun N-terminal kinase (JNK) to participate in cell migration and cell polarization. JNK subsequently activates transcription factors such as Jun proto-oncogene (c-Jun) and activator protein 1 (AP-1) to regulate downstream gene expression. RhoA is involved in activation of RHO-associated kinase (ROCK), which regulates actin polymerization and cytoskeleton remodeling (Fig. 1).

In the Wnt/Ca^2+^ signaling pathway, the complex formed by FZD and RYK or ROR1/2 can also activate phospholipase C (PLC) by activating G proteins on the cell membrane after binding to Wnt proteins. Activated PLC can decompose phosphatidylinositol-4,5-bisphosphate (PIP2) into 1,2-diacylglycerol (DAG) and inositol-1,4,5-trisphosphate (IP3) on the cell membrane. IP3 induces the release of large amounts of Ca^2+^ into the cytoplasm through IP3-sensitive Ca^2+^ channels in the endoplasmic reticulum. At this point, the decrease of intracellular Ca^2+^ store leads to the opening of Ca^2+^ channels regulated by Ca^2+^ reservoirs on the cell membrane, which causes a large influx of Ca^2+^ into the cytoplasm, resulting in a transient increase in free cytoplasmic Ca^2+^. In concert with Ca^2+^, DAG can also activate protein kinase C (PKC), which activates CDC42 to participate in morphological maturation processes such as cytoskeleton remodeling. Ca^2+^ can simultaneously activate calcineurin, which once activated can dephosphorylate nuclear factor-activated T cell (NFATc) protein. Subsequently, the dephosphorylated NFATc protein is transferred to the nucleus to form NFAT complexes (NFATn) with specific transcription factors to regulate the expression of downstream target genes. In addition, Ca^2+^ activates calmodulin-dependent protein kinase II (CaMKII), which phosphorylates transforming growth factor (TGF)-β-activated kinase 1 (TAK-1) to induce activation of the transcription factor Nemo-like kinase (NLK). NLK can act as an inhibitor of the canonical Wnt signaling pathway by reducing binding of β-catenin to TCF/LEF [100] (Fig. 1).

## Effect of high glucose on non-canonical wnt signaling in bone homeostasis

### Ligands

Although Wnt3a is usually considered a ligand for activation of the canonical Wnt signaling pathway, studies have shown that it can also mediate osteoblastogenesis independently of β-catenin by activating the heterotrimeric G protein G_α(q/11)_/PLC/PKC pathway in ST2 cells or JNK pathway in human mesenchymal stem cells (hMSCs) [101, 102]. However, Wnt3a expression was significantly decreased in a mouse model of type 2 diabetic osteoporosis [48], suggesting that the reduction of Wnt3a in a high-glucose state could inhibit bone formation by affecting the non-canonical Wnt signaling pathway in these cells. Insulin can promote bone formation. As a ligand of the non-canonical Wnt signaling pathway, Wnt4 can completely antagonize Wnt3a-stimulated cell growth and insulin secretion in a rat islet cell tumor cell line (INS-1) [103]. However, Wnt4 expression in islet cells of individuals with diabetes is increased, leading to enhancement of the JNK pathway in Wnt/PCP signaling, which impairs β cell proliferation and insulin secretion [104]. Thus, the increase of Wnt4 in individuals with diabetes can indirectly affect the osteogenesis process by affecting insulin synthesis (Fig. 4).

Wnt5a can increase osteoblast differentiation by activating the Ror2/ROCK pathway in hMSCs [105]. However, Xu et al. observed a trend for increased Wnt5a levels in individuals with long-term T2DM or T2DM after 3 months of treatment. Accordingly, the authors suggest that this may be one of the reasons for the elevated BMD in individuals with T2DM [106]. However, bone strength and turnover are low in individuals with T2DM [107] due to osteoblast-expressed Wnt5a accelerating the differentiation of monocytes into osteoclasts [108]. Furthermore, in osteoclast precursors, Wnt5a also promotes the binding of RANKL to receptor activator of nuclear factor-κB (RANK) by binding to ROR2 [109]. Although the binding of RANKL to RANK will sequentially activate tumor necrosis factor-associated factor 6 (TRAF6) and nuclear factor (NF)-κB, the latter can regulate osteoclast formation by activating downstream NFATc1. However, Zhang et al. found that the canonical Wnt signaling pathway was significantly inhibited in a rat model of type 2 diabetic osteoporosis, while the RANKL-activated TRAF6/NF-κB/NFATc1 pathway was significantly activated, resulting in increased osteoclast differentiation [110]. These results suggest that high glucose can also promote bone resorption through this pathway. Razny et al. observed increased expression of RANK and RANKL in obese individuals with T2DM and insulin resistance [66]. RANKL levels were also significantly elevated in children and adolescents with T1DM [111]. Ding et al. confirmed that high glucose could enhance the differentiation of RAW264.7 cells into osteoclasts by increasing the RANKL/OPG ratio. Moreover, the enhancement of bone resorption was caused by high glucose inducing the production and accumulation of AGEs in vivo [112]. After applying a combination of high glucose plus palmitate to mimic the effects of the microenvironment of T2DM on osteoclast differentiation, Qu et al. found that this environment promoted differentiation of osteoclast precursors into osteoclasts by decreasing OPG expression and increasing levels of RANK, RANKL, and NFATc1. Moreover, this study found that attenuating the activity of p66Shc both inhibited ROS production and reversed the increase in osteoclastogenesis caused by high glucose and palmitate [113]. In summary, although elevating Wnt5a in a hyperglycemic state can increase BMD, this alone is insufficient to fully assess the risk of fracture in individuals with diabetes [114]. In addition, elevated Wnt5a enhances the binding of RANK to RANKL, leading to increased osteoclast production. Hyperglycemia can decrease expression of OPG, the main decoy receptor of RANKL, leading to an increase in RANKL. Therefore, the authors concluded that the elevation of Wnt5a caused by long-term hyperglycemia will eventually elicit a negative balance of bone homeostasis (Fig. 5).

Keats et al. demonstrated that high glucose significantly increased expression of Wnt11, another ligand of the non-canonical Wnt signaling pathway, in human bone marrow mesenchymal progenitor cells (MPCs), which promoted their differentiation towards adipocytes rather than osteoblasts through the Wnt11/PKC pathway [115]. Wnt16 promoted osteoblast formation through the transcription factors recombinant FOS-like antigens 1 and 2 (Fosl1 and Fosl2), which are activated by the non-canonical Wnt/JNK pathway in primary osteoblasts [116]. Wnt16 not only inhibits osteoclastogenesis by blocking the binding of RANKL to RANK, it reduces osteoclast differentiation by inhibiting RANKL-induced activation of NF-κB and NFATC1 by activating the non-canonical Wnt signaling pathway in osteoclast precursors. In addition, Maeda et al. observed normal bone mass of cancellous bone and significantly reduced bone mass of cortical bone in *Wnt16*-knockout mice, indicating that Wnt16 expression in cortical bone was higher than in cancellous bone [117]. However, compared with that in the control group, Wnt16 expression was significantly reduced in a type 1 diabetic osteoporosis mouse model, leading to a significant increase in the cortical bone porosity and bone resorption. Mechanistically, the accumulation of oxidative stress caused by hyperglycemia was implicated in these observed abnormalities [53] (Figs. 4 and 5). Notably, the findings of this study also explain the increased presence of cortical bone defects [89] in adults with T1DM.

### Receptors

Both ROR1 and ROR2 are essential for bone development. However, the prevailing view is that only ROR2 affects bone mass. Maeda et al. demonstrated that Wnt5a-ror2 signaling in osteoclast precursors can enhance RANKL-induced osteoclastogenesis by activating JNK and recruiting c-Jun to the *RANK* gene promoter [117]. However, there is a lack of studies on the effect of ROS on ROR2 expression in osteoclast precursors. Lee et al. showed that ROS can cause periodontitis by upregulating expression of Wnt5a, FZD, and ROR2 in human periodontal ligament cells [118]. As mentioned above, Wnt5a expression is increased in individuals with diabetes. Therefore, ROS produced in response to high glucose appears to activate Wnt5a-ROR2 signaling in osteoclast precursors to increase osteoclast differentiation.

### SFRPs and WIF1

Although SFRP4 can reduce osteoclast formation and reduce osteoclast activity by inhibiting the non-canonical Wnt/Ror2/JNK cascade [119], Haraguchi et al. demonstrated that persistent elevation and activation of SFRP4 could unlock the inhibitory effect of canonical Wnt signaling on osteoclastogenesis, resulting in low bone mass [94]. In addition, SFRP4 has been be closely associated with insulin resistance and the progression of T2DM. SFRP4 expression is significantly higher in patients with prediabetes, T2DM, or gestational diabetes compared with that in healthy individuals [120–122]. Mori et al. also found that treatment of ST2 cells with methylglyoxal (a glucose intermediate metabolite) rapidly enhanced SFRP4 expression and inhibited expression of osteogenic Wnt-targeting genes, including OPG [95]. Taken together, the elevation of SFRP4 caused by high glucose ultimately has a detrimental effect on bone formation (Fig. 5).

WIFI also inhibits non-canonical Wnt signaling pathway. WIF1 is elevated in individuals with T2DM [66], however, there is a lack of researches related to WIF1 affecting osteoblastogenesis, osteoclastogenesis, or adipocytogenesis through non-canonical Wnt signaling. Therefore, we believe that exploring high glucose to affect bone remodeling via non-canonical Wnt signaling, WIF1 will have the potential to be one of the key factors in the future.

### Wnt/planar cell polarity signaling pathway

Although differentiation of osteoblasts requires involvement of the RhoA/ROCK pathway [123], its activation can have a significant negative regulatory effect on osteoblastogenesis [124]. Moreover, activation of the RhoA/ROCK pathway inhibits osteoclast formation by osteoclast precursors [125]. However, there is a lack of data describing the effects of the RhoA/ROCK pathway on osteoblastogenesis and osteoclastogenesis in a high-glucose state. Cheng et al. showed that high glucose induces activation of the RhoA/ROCK pathway in RAW264.7 cells [126]. Activation of the RhoA/ROCK pathway is an important cause of long-term complications of diabetes [127]. Therefore, high glucose may activate the RhoA/ROCK pathway in both osteoblast and osteoclast precursors, thereby inhibiting bone formation and resorption; however, more studies are needed. It is certain that restoring the normal functioning of RhoA/ROCK by controlling hyperglycemia can be an important target for the treatment of diabetic complications, including diabetic osteoporosis.

Rac1 and Cdc42 are involved in bone resorption processes such as osteoclast polarization and inhibition of osteoclast apoptosis. Umbayev et al. [128] demonstrated that inhibition of Cdc42 levels increased differentiation of rat adipose-derived mesenchymal stem cells into osteoblasts and decreased ROS levels in these cells. When RANKL-induced osteoclast differentiation and bone resorption are inhibited, the activities of Rac1 and JNK are simultaneously diminished [129], suggesting a positive feedback effect of the Rac1/JNK cascade in osteoclastogenesis and role in bone resorption. In general, ROS is considered a pathological factor in inflammation and aging. For example, Lee et al. found that RANKL-stimulated BMMs transiently increased intracellular ROS levels by activating signaling cascades, including Rac1 and JNK, and demonstrated that ROS can act as a physiological second messenger in RANKL signaling to promote osteoclast formation [130]. Specifically, Rac1 was closely associated with ROS production [131]. Thus, ROS produced by high glucose can ultimately increase osteoclast differentiation by activating the Rac1/JNK cascade in osteoclast precursors (Fig. 5). Rac1 is also important for the nuclear translocation of β-catenin during osteoblastogenesis. Although there is a lack of data regarding the effect of high glucose on Rac1 in osteoblast precursors or immature osteoblasts, it has been shown that silencing Rac1 limits ROS production to protect cells from ROS-induced damage and apoptosis [132]. Accordingly, Rac1 may also be an important therapeutic target for reducing osteoblast damage caused by high glucose-induced oxidative stress. Activation of the JNK pathway has a facilitative effect on differentiation of hMSCs into osteoblasts and inhibits adipocyte production [133]. However, Wnt signaling factors associated with the non-canonical pathway (i.e., Wnt5a/b, Ror1, Ror2, whole and phosphorylated JNK, whole and phosphorylated c-Jun) were almost constant in ST2 cells treated with methylglyoxal compared with those in control ST2 cells [95]. Furthermore, Chaves et al. demonstrated that high glucose failed to increase JNK phosphorylation in BMSCs from young Wistar and spontaneous hypertensive rats without hypertension. However, the concentration of glucose used in the experiment was insufficient to cause more severe damage to BMSCs, which the authors note may have contributed to the results of the study [134]. Thus, the effect of high glucose on the Rac1/JNK pathway in osteoblast precursors and immature osteoblasts needs further investigation.

### Wnt/Ca^2+^ signaling pathway

Strack et al. found that the G_α(q/11)_/PLC/PKC pathway is similar to the RANKL/RANK pathway, and both can promote the differentiation of osteoclast precursors into osteoclasts [135]. The G_α(q/11)_/PLC/PKC pathway can promote differentiation of ST2 cells into osteoblasts. ROS produced by high glucose has been found to activate PKC by increasing free Ca^2+^ concentrations in the cytoplasm, thereby enhancing bone morphogenic protein 2-induced collagen synthesis and early osteogenic gene expression in rat spinal ligament cells [136]. However, because there is a current lack of studies on the effects of high glucose on bone formation or bone resorption through this pathway, it represents a new research direction for diabetic osteoporosis.

The Ca^2+^/CaMKII/TAK-1/NLK pathway inhibits the transactivation of peroxisome proliferator-activated receptor γ and induces Runx2 expression, thereby promoting the differentiation of bone marrow mesenchymal progenitors towards osteoblasts rather than adipocytes. However, Li et al. found that NLK expression was significantly increased in BMSCs from type 2 diabetic Goto-Kakizaki rats, which in turn triggered TCF inactivation to inhibit the bone formation process induced by the canonical Wnt signaling pathway. Again, the reported abnormalities were closely associated with the production of AGEs and insulin resistance [137] (Fig. 4). Ca^2+^ concentrations and CaMKII phosphorylation were significantly increased in osteoclasts differentiated from RAW264.7 cells treated with macrophage colony-stimulating factor and RANKL [138], implicating the Ca^2+^/CaMKII pathway in osteoclast differentiation. However, Shen et al. found that hyperglycemia-induced oxidative stress can increase osteoclastogenesis and bone resorption in type 1 diabetic rats by increasing free Ca^2+^ concentrations in BMMs, thus activating the Ca^2+^/CaMKII pathway. Moreover, application of KN93 (a CaMKII inhibitor) significantly alleviated the hyperglycemia-induced abnormal bone morphology in rats but failed to reduce hyperglycemia and body weight [139]. Activation of CaMKII can stimulate the TAK-1/NLK pathway. Thus, based on the information above, it can be concluded that high glucose can induce activation of the Ca^2+^/CaMKII/TAK-1/NLK pathway in osteoclast precursors and increase SFRP4 expression. The result is more severe inhibition of the Wnt/β-catenin pathway in these cells, which increases osteoclast formation to further aggravate bone resorption (Fig. 5).

The Ca^2+^/Calcineurin/NFATc1 pathway plays a key role in the negative regulation of osteoblast differentiation. Vadavanath et al. showed that ROS can activate the Ca^2+^/calcineurin/NFATc1 pathway to regulate the expression of specific transcription factors [140]. However, Jie et al. found that application of FK506 (a calcineurin inhibitor) significantly alleviated the detrimental effects of oxidative stress on bone formation by activating the Ca^2+^/Calcineurin/NFATc1 pathway in MC3T3-E1 cells [141]. These results indicate that in a high-glucose state, ROS production activates the Ca^2+^/Calcineurin/NFATc1 pathway in osteoblast precursors to inhibit their differentiation into osteoblasts (Fig. 4). Moreover, the Ca^2+^/calcineurin/NFATc1 pathway is particularly critical in the process of osteoclast differentiation [142]. In this pathway, NFATc1 acts a key factor for osteoclast differentiation; indeed, even in the absence of osteoclast differentiation factors, ectopic overexpression of NFATc1 is sufficient to induce osteoclast differentiation [143]. However, Kim et al. found that ROS can promote the differentiation of bone marrow-derived monocytes into osteoclasts by enhancing the PLCγ/Ca^2+^/NFATc1 pathway [144]. Collectively, these results suggest that increased ROS production in response to high glucose can accelerate osteoclast differentiation by activating the PLCγ/Ca^2+^/NFATc1 pathway in osteoclast precursors (Fig. 5).

Based on the findings described above, we conclude that abnormalities of the non-canonical Wnt pathway will affect the morphological maturation of many cell types. Hyperglycemia not only disturbs this pathway by affecting the expression of ligands and receptors, it can directly interfere with the PCP or Ca^2+^ pathways of these cells by producing ROS and AGEs. Thus, if hyperglycemia is prolonged, the effects on affected cells are more harmful and inhibiting CaMKII or calcineurin activity may be a new therapeutic target for diabetic osteoporosis.

## Conclusion

Therefore, it can be concluded that high glucose affects bone homeostasis by disrupting the Wnt pathway. In addition, high glucose can interfere with the normal bone remodeling process by affecting multiple cell types simultaneously. In this process, production of ROS and AGEs, as well as the development of insulin resistance, are critical disruptors of the Wnt pathway. Hyperglycemia can inhibit bone formation and promote bone resorption by suppressing the canonical Wnt signaling pathway. In addition, the non-canonical Wnt signaling pathway can indirectly lead to impaired bone formation by inhibiting insulin secretion and promoting the differentiation of MPCs into adipocytes. Compared with the canonical Wnt pathway, alterations in levels of other Wnt ligands, the degree of Ror2 receptor expression, and interference with the Wnt/PCP pathway by hyperglycemia have not been studied enough. Therefore, more attention should be paid in the future to the effects of hyperglycemia on bone homeostasis through the non-canonical Wnt pathway, and more clinical trials should be conducted accordingly. This strategy will bring about more possibilities for designing new drugs to treat diabetic osteoporosis by interfering with the non-canonical Wnt signaling pathway.

## Data Availability

Not applicable.
